# Differential modulation of the auditory steady state response and inhibitory gating by chloral hydrate anesthesia

**DOI:** 10.1038/s41598-018-21920-x

**Published:** 2018-02-27

**Authors:** Yuchen Wang, Lanlan Ma, Xuejiao Wang, Ling Qin

**Affiliations:** Department of Physiology, College of Basic Medical Science, China Medical University, No. 77 Puhe Road, Shenyang North New Area, Shenyang, Liaoning Province 110122 P. R. China

## Abstract

Auditory steady state response (ASSR) and inhibitory gating (IG) are electrophysiological examinations commonly used to evaluate the sensory and cognitive functions of the brain. In some clinic examinations and animal experiments, general anesthesia is necessary to conduct electrophysiological recordings. However, the effects of anesthesia on ASSR and IG remain unclear. For this reason, we recorded local field potentials though electrodes implanted in different brain areas of rats: the auditory cortex (AC), hippocampus (HC), amygdala (AMY), and prefrontal cortex (PFC), and compared the characteristics of ASSR and IG under anesthetized and conscious conditions. We found that ASSR signals were the strongest in the AC, and decreased sequentially in the HP, AMY, and PFC. Chloral hydrate anesthesia significantly reduced the power and phase-locking of ASSR in the AC, HP, and AMY. In contrast, the extent of IG in the AC was weakest and it increased sequentially in the HP, AMY, and PFC. Anesthesia had less effect on the extent of IG. Our results suggest that ASSR and IG may originate from different neural circuits and that IG is more resistant to general anesthesia and therefore better suited to examining the functioning of non-auditory brain regions.

## Introduction

Auditory steady state response (ASSR) and inhibitory gating (IG) are commonly used to evaluate the sensory and cognitive functions of the central nervous system. Electroencephalographic (EEG) signals entrained to periodic auditory stimuli (a train of clicks) are often referred to as the ASSR. The power (magnitude) and phase locking ability (phase consistency across trials) of the ASSR can reflect the functional integrity of the neural circuits that support synchronization across frequencies^1,2^. EEG measurement of ASSR, particularly in the gamma frequency range (30–80 Hz), has been commonly used in the clinical examination of mental illness^3–8^ and in neuropharmacological experiments in animal models^9,10^.

IG is experimentally characterized by a reduced responsiveness to redundant stimuli. IG is estimated using evoked response potential (ERP) techniques in which identical pairs of auditory stimuli (tones, white noises or clicks) are presented and ERP responses to the stimuli are elicited and then compared. The first stimulus in a pair is commonly identified as the conditioning stimulus (C) and the second one is called the test stimulus (T). The C and T stimuli are separated by an inter-trial interval of 0.5 s, and trial pairs are typically separated by 8–10 s^11–14^. In general, the amplitude of the response to T stimuli is lower than that to C stimuli^15^, the ratio of responses to T and C theoretically represents IG^16^. IG has been proposed as a pre-attentional mechanism involved in sensory information processing and the modulation of responses to stimuli^15,17^. Healthy IG function is thought to promote the filtering of irrelevant information from important sensory stimuli^18,19^. Examination of IG may reveal some dysfunctions of brain^11,19–22^.

Though ASSR and IG have been widely used, some methodological issues remain unclear. Because general anesthesia is required in some cases of clinical examination and animal experimentation, the effects of anesthesia on ASSR and IG warrant investigation. Furthermore, the exact anatomical sites involved in mediating ASSR and IG in humans have not yet been established due to the limitations of performing studies using intra-cerebral electrodes. Studies in animals can provide more insight into the areas of the brain involved in ASSR and IG. Therefore, we designed this study to examine the effects of anesthesia on ASSR and IG recorded from different brain regions. We implanted microelectrodes in the auditory cortex (AC), hippocampus (HC), amygdala (AMY), and prefrontal cortex (PFC) of rats and recorded the local field potentials (LFPs) evoked by auditory stimuli (click-trains and noise pairs) under conscious and chloral hydrate anesthetized conditions. We selected the HP, AMY, and PFC because they play vital roles in advanced brain functions (memory, emotion, and motivation). By comparing the LFPs recorded from the auditory and non-auditory regions, we found different anatomical distributions of ASSR and IG, and different effects of anesthesia on ASSR and IG.

## Materials and Methods

### Animals

All experimental protocols were approved by the China Medical University Animal Care and Use Committee and were in strict accordance with the National Institutes of Health Guide for the Care and Use of Laboratory Animals (NIH Publications No. 80-23) revised in 1996. All surgeries were conducted under anesthesia and maximum efforts were taken to ameliorate animal suffering. Adult male Wistar rats weighing 250–300 g (8–10 weeks old) were used for the experiments. Animals came from our own colony housed in a humidity-controlled (50–55%) and temperature-controlled (22–24 °C) facility and were on a 12-h light/dark cycle (lights on at 7:30 A.M.) with ad libitum access to food and water.

### Surgical preparation and electrode implantation

The animal was anesthetized by an intraperitoneal injection of urethane (1.5 g/kg). Supplementary doses (0.5 g/kg) were given as needed. Atropine sulfate (0.1 mg/kg) was used to reduce the viscosity of bronchial secretions. Temperature was monitored rectally and maintained at 37 °C using a feedback-controlled blanket. After placing the animal in a stereotaxic frame (SR-5R, Narishige, Tokyo, Japan), the cranium was exposed. Four stainless steel guide tubes (27 G) were separately inserted into the left hemisphere of the AC (A −4.0, M +3.0, D −1.5), HP (A −5.2, M +5.4, D −3.8), AMY (A −3.0, M +4.9, D −8.5), and PFC (A +4.8, M +0.5, D −4.0) according to the standard rat stereotaxic atlas^23^. After fixing the tubes using dental acrylic, we lowered a Teflon-coated sliver microwire (#78550, A-M Systems, USA) into each tube and kept the tip of the microwire at the level of 0.5 mm below the opening of the tube. The other end of the microwire was soldered to a pin connector, which was secured onto the cranium of the right hemisphere using dental cement. The stainless guide tubes implanted in the AC, HP, AMY, and PFC served as references.

### Sound presentation system and auditory stimulus

Sound stimuli were generated by custom-built programs in the MATLAB (The Mathworks, Nantic, MA, USA) environment and delivered via an earphone (NW-STUDIO PRO W; Ninewave, Japan), which was attached to the cement platform implanted on the rat’s skull during the surgery. The placement of the earphone was adjusted to 1 cm from the ear canal contralateral to the recording side. We used a train of click sounds to assess ASSR. The waveform of each click was a rectangular pulse of a 0.2-ms duration, which repeated at a rate of 40 cycles/s and continued for a 0.5-s duration. In each session, the click-trains were presented 120 times with inter-train-intervals of 2–4 s. After the recording of ASSR, 120 pairs of sounds were presented to the rats to evaluate IG. The sound pairs consisted of two 80-ms sounds of white noise (5-ms rising/falling time), presented at a fixed interval of 500 ms. The first sound in a sound pair was identified as the conditioning sound (C) and the second one was called the testing sound (T). There was an 8–10-s interval between the pairs of sounds. The peak of all the sound waves was adjusted to the level of a 4k-Hz pure-tone at a 55-dB sound pressure level, measured by placing a sound pressure meter (Brüel & Kjaer, 1/2-inch condenser microphone with a pre-amplifier 2669) 1 cm in front of the earphone.

### Recording procedure

Electrophysiological recording was conducted in an electrically shielded, soundproof box. Before the recording experiments, the rats were acclimated to handling and to the laboratory for 5 days. The rats were habituated to the recording box and sound stimuli for 30 min on day 5. On the recording day, a flexible, low noise cable was connected to the pin connector implanted on the skull of the rat. The microwire output was delivered to a multi-channel preamplifier (RA16PA; TDT, Alachua, FL, USA), then to a digital signal processing module (RZ-2; TDT). The waveforms of the field potentials were obtained by applying a bandpass filter (1–300 Hz). The field potentials were imported into Matlab for further analysis. First, we recorded the LFPs evoked by click-trains and sound pairs when the rats were free (conscious condition). The recording session lasted about 30 min. Then, the rats were anesthetized with chloral hydrate (330–350 mg/kg, 5%, i.p.). Twenty minutes later, when the rats were under general anesthesia indicated by the disappearance of nociceptive reflex (anesthetized condition), the LFPs evoked by click-trains and sound pairs were recorded again. The recording procedure was repeated 2–4 times on each rat with an interval of at least 3 days.

### Analysis of LFP

LFPs evoked by a 40-Hz click-train were analyzed using a wavelet-based analysis algorithm, implemented in custom written Matlab scripts, to obtain their mean trial power (MTP) and phase-locking factor (PLF)^24^. MTP was computed by averaging the LFP power in the spectral-temporal domain across the 120 trials from one session. PFL measures the synchronization of the LFP phase across individual trials at particular frequencies and time intervals.

LFPs evoked by sound pairs were quantified through amplitude measurements for certain negative and positive peaks in the waveform averaged across the 120 recording trials. The peaks were identified according to the local maxima (or minima) in predefined time windows, and the peaks were measured according to the difference in peak amplitude (positive or negative) from the baseline amplitude at the time of stimulus onset. With sliding-window t-tests, the LFP peaks were compared with activity during a 1-s control period 3 s before each C stimulus. Only the LFP peaks that differed from the control period at the 0.01 level of significance were used for further analysis. The responses that differed significantly from the control period were designated as C-amp or T-amp, respectively, for the amplitude of response to C or T. The ratio of T-amp/C-amp (T/C) served as a crucial comparison for IG.

### Data availability

The datasets generated during and analyzed during the current study are available from the corresponding author on reasonable request.

## Results

We implanted the microwires on the left-brain hemisphere of 34 rats and conducted 98 recording sessions. In total, we successfully acquired 93 sound-evoked LFP data sets in the AC, 74 in the HP, 68 in the AMY, and 64 in the PFC.

### Effect of anesthesia on ASSR

**Figure 1:**
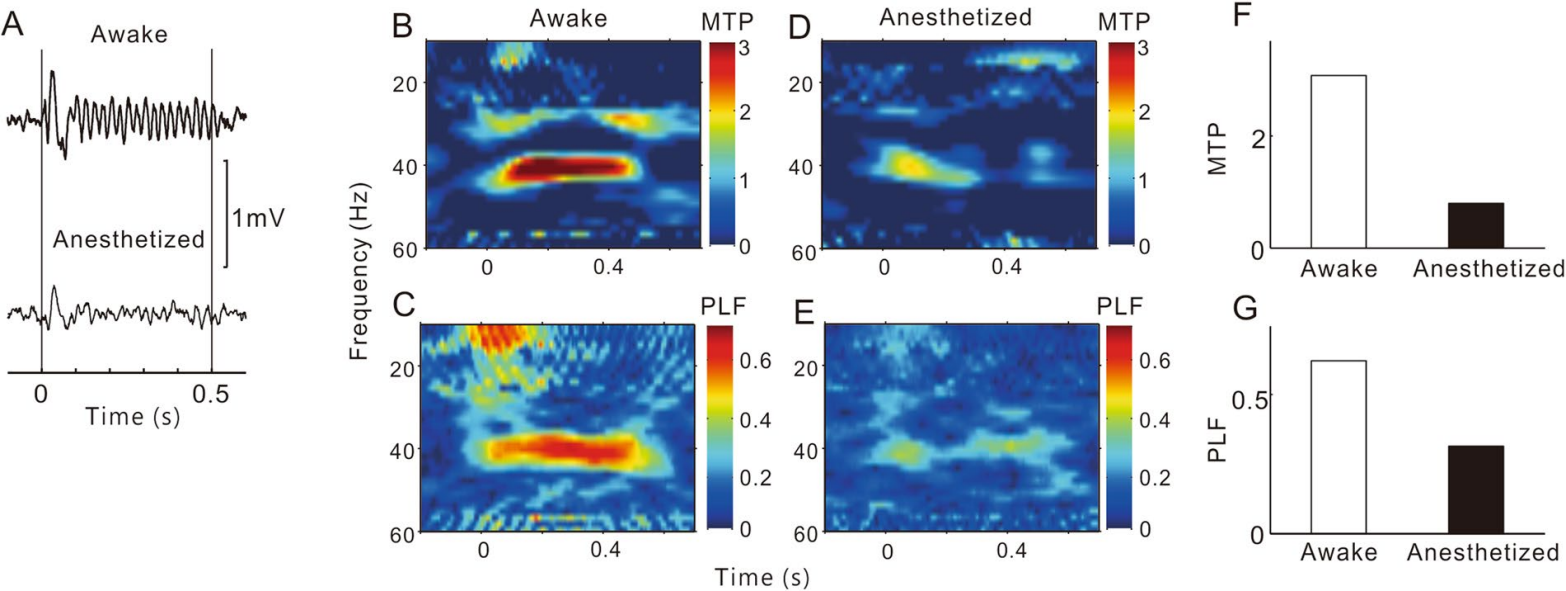
A representative example of ASSR in AC sites. (**A**) LFPs evoked by click-train stimulation. Vertical lines mark the onset and offset of the click-trains. (**B**) and (**C**) Time-frequency plot of MTP and PLF under conscious conditions. (**D**) and (**E**) Time-frequency plot of MTP and PLF under anesthetized conditions. (**F**) and (**G**) the value of MTP and PLF at 40 Hz under conscious and anesthetized conditions.

Under conscious conditions, click-train stimulation evoked robust and time-locked oscillatory changes in the LFP of the AC, as shown by the example in Fig. 1A. We used time-frequency analysis to quantitatively evaluate the MTP and PLF. MTP reveals the amplitude of the evoked LFP, and PLF examines the trial-to-trial dynamics of the phase-locked response. The time-frequency plots of MTP and PLF in response to a 40-Hz stimulation are shown in Fig. 1B and C. It is clear that the LPF of this site showed strong MTP and PLF at 40 Hz. Under anesthetized conditions, the oscillation of the LFP waveform and the strength of the MTP and PLF became smaller (Fig. 1D,E). The mean MTP (averaged across a 35–45-Hz frequency range and a 50–550-ms time window) and PLF of the conscious and anesthetized conditions are presented in Fig. 1F and G, and indicate that the MTP and PLF were obviously reduced by anesthesia.

**Figure 2:**
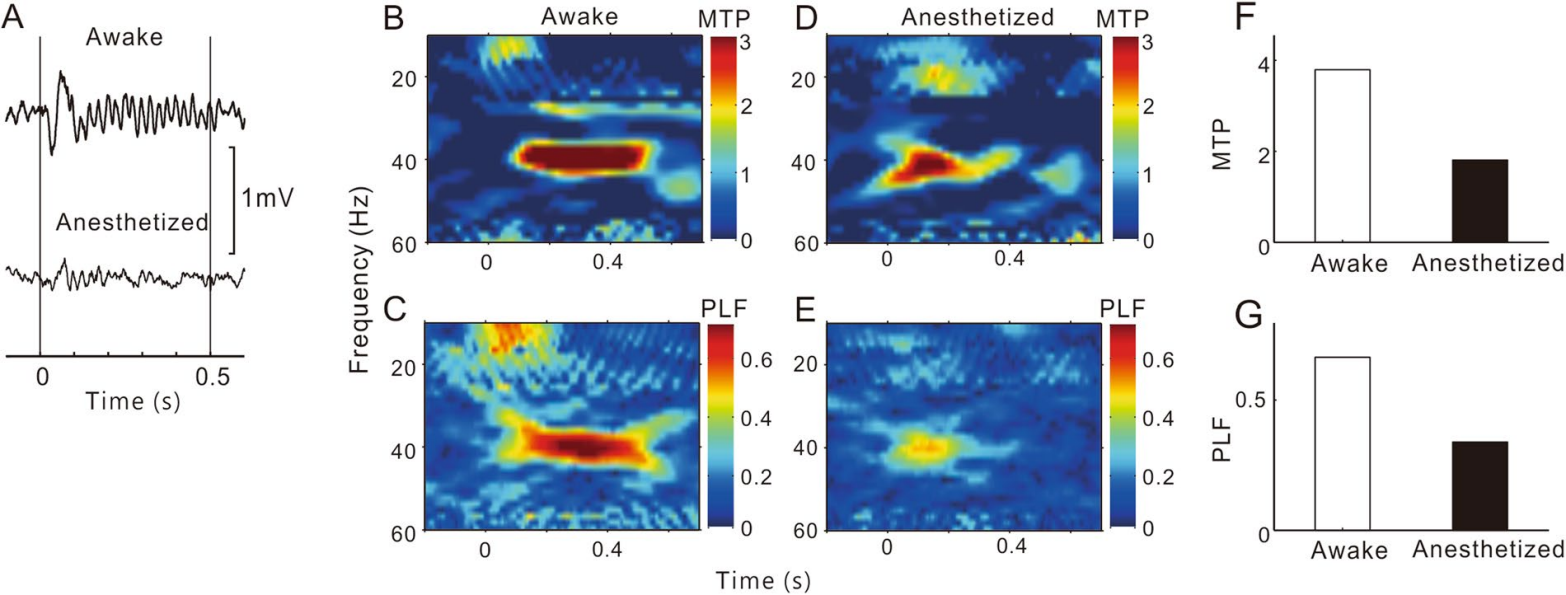
A representative example of ASSR in HP sites. Same format as Fig. 1.

**Figure 3:**
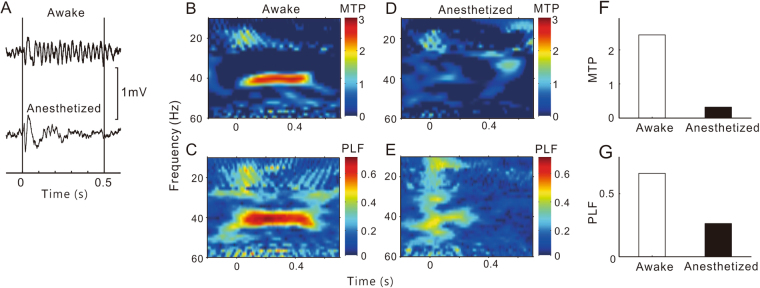
A representative example of ASSR in AMY sites. Same format as Fig. 1.

**Figure 4:**
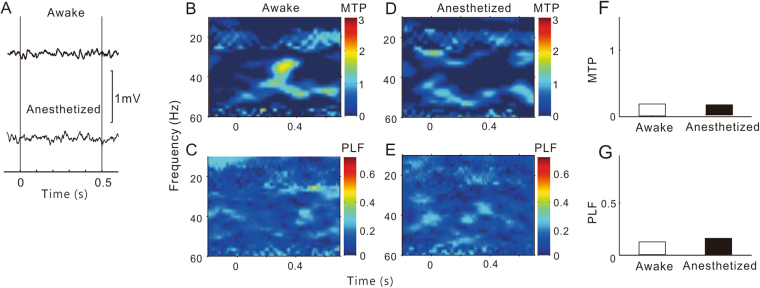
A representative example of ASSR in PFC sites. Same format as Fig. 1.

The representative results for the HP, AMY, and PFC sites are presented in Figs 2, 3 and 4, respectively. The LFPs in the HP and AMY showed a clear ASSR under conscious conditions, which was largely suppressed under anesthetized conditions. In the PFC site, we could not record a robust ASSR under any conditions.

**Figure 5:**
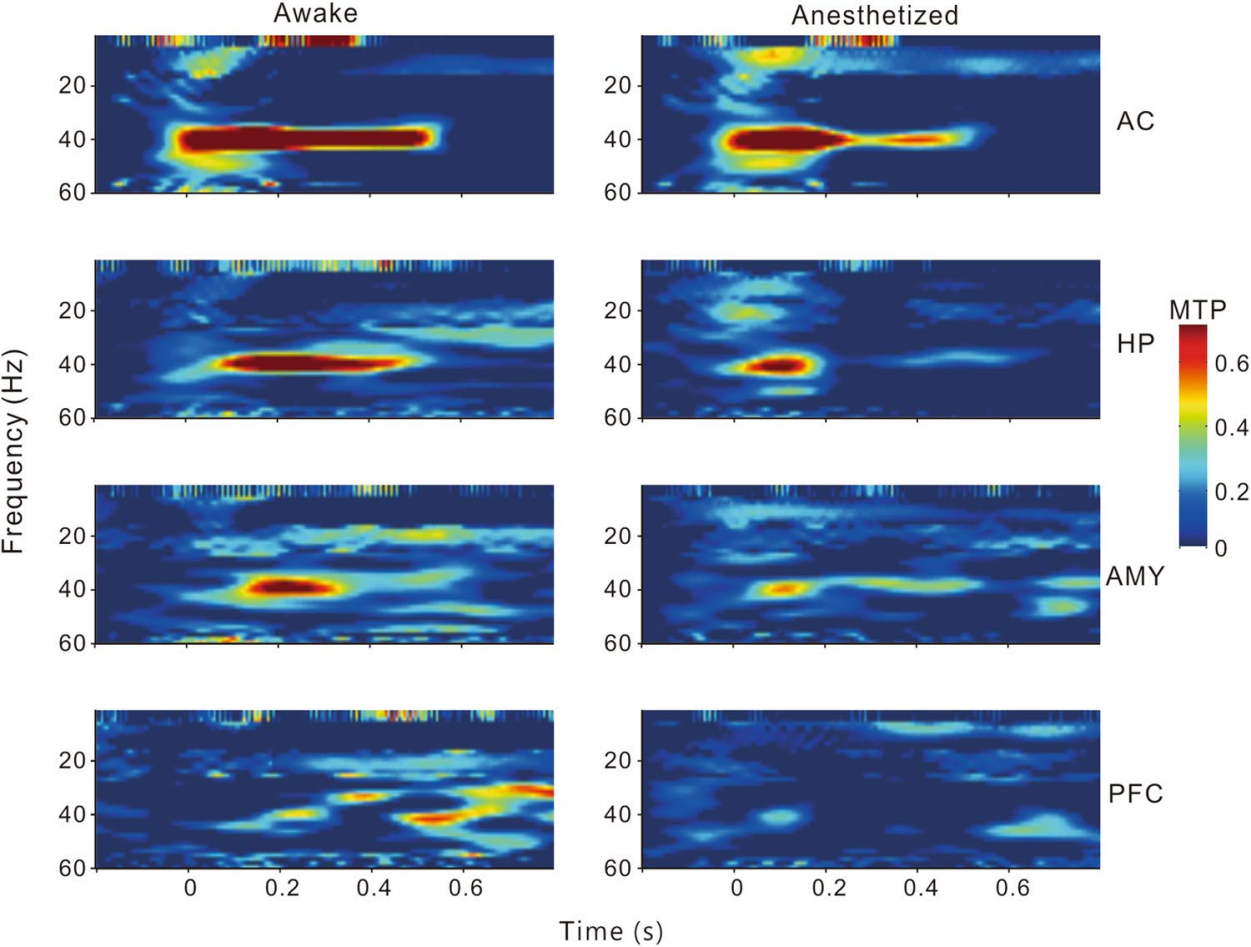
Time-frequency plot of MTP averaged across each brain region.

**Figure 6:**
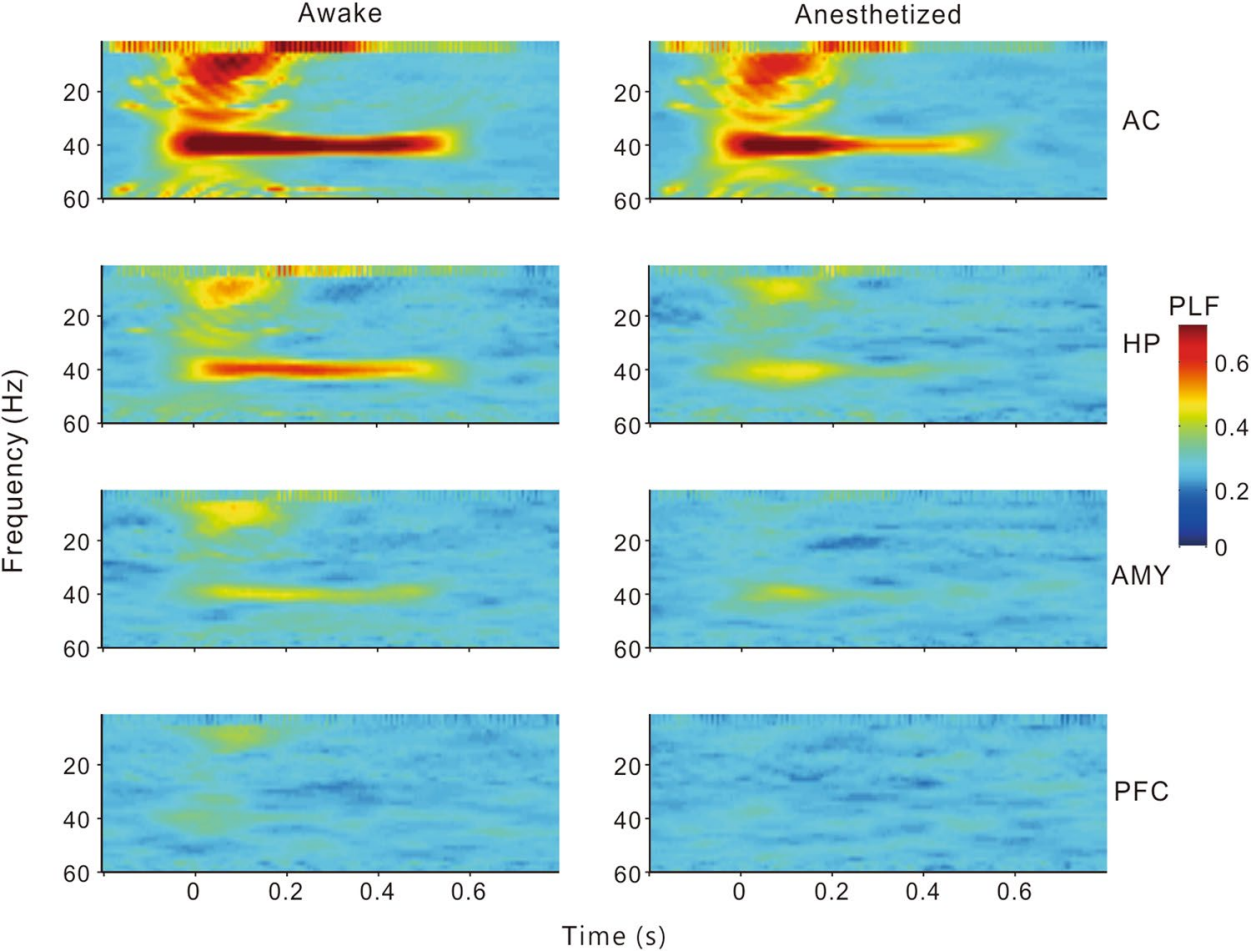
Time-frequency plot of PLF averaged across each brain region.

**Figure 7:**
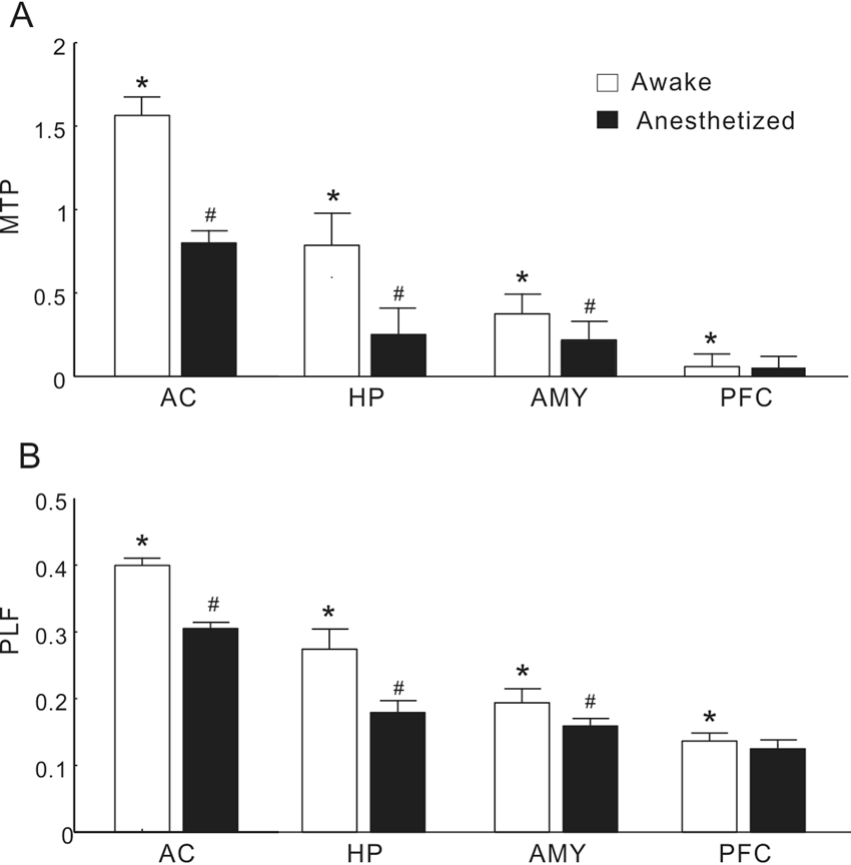
MTP (**A**) and PLF (**B**) under conscious and anesthetized conditions in different brain regions. The data are expressed as the mean ± SE. *Represents a significant difference comparing to any other brain regions under conscious conditions (P < 0.05, ANOVA with Tukey’s post hoc tests). ^#^Represents a significant difference between conscious and anesthetized conditions in each brain region (P < 0.05, paired t-test).

To compare the population results of different brain regions, we constructed mean time-frequency plots of MTP and PLF averaged across all the individual sites in the AC, HP, AMY, and PFC (Figs 5 and 6). It is clear that under conscious conditions the MTP and PLF were strongest in the AC, gradually decreasing in the HP and AMY, and disappearing in the PFC. Under anesthetized conditions, the MTP and PLF reduced correspondingly. Statistical analysis of the 40-Hz MTP and PLF revealed a significant difference between different brain regions (ANOVA and Tukey’s multiple comparison, p < 0.05, Fig. 7) and between the conscious and anesthetized conditions (paired t-test, p < 0.05, Fig. 7). In summary, different brain regions showed different degrees of ASSR, all of which were reduced by anesthesia.

### Effect of anesthesia on IG

**Figure 8:**
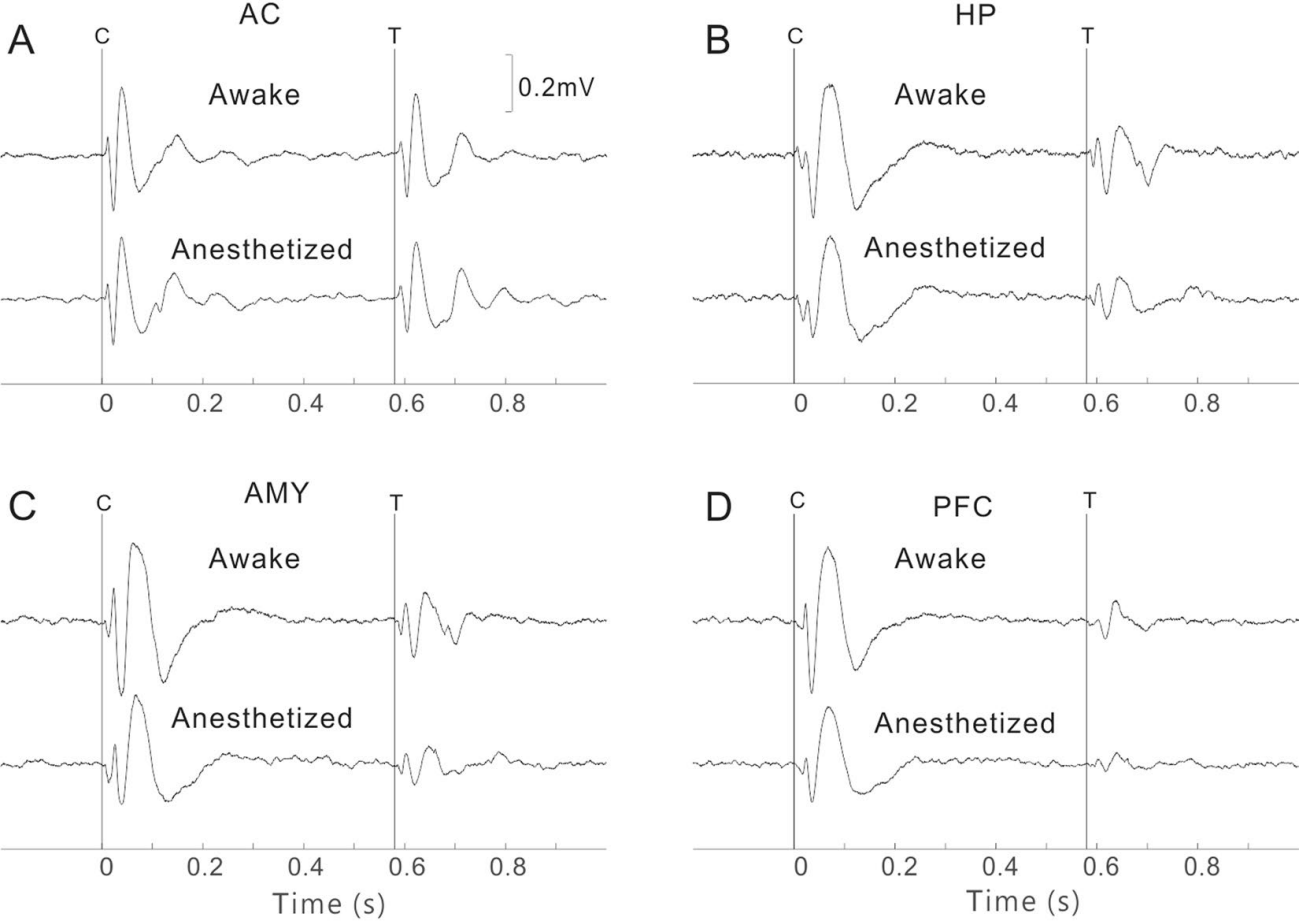
LFPs evoked by sound pairs under conscious and anesthetized conditions in the AC (**A**), HP (**B**), AMY (**C**), and PFC (**D**). Vertical lines represent the onset of C and T stimuli.

Figure 8A shows an example of the LFP evoked by sound pairs in the AC. Under conscious conditions, the amplitude of LFP evoked by T stimulus was smaller than that evoked by C stimulus, indicating an IG effect. We quantified the LFP by measuring the mean negative deflection at about 25 ms (N25) after the stimulus onset, and the mean positive deflection at about 40 ms (P40). The T/C ratio of N25 was 0.75 and that of P40 was 0.90. A T/C ratio < 1.0 reflects an IG effect. Under anesthetized conditions, the absolute amplitudes of LFP evoked by both C and T stimulus were reduced a little, but the T/C ratio of N25 and P40 remained similar (0.72 and 0.92). Examples of LFP in the HP, AMY, and PFC are presented in Fig. 8B,C and D, respectively. Under conscious conditions, the T-amp (the amplitude of T-evoked deflection) showed a remarkable tendency to decrease progressively as the recording site moved from the AC to the PFC. Another noteworthy result is that the latencies of evoked deflection were longer in the HP, AMY, and PFC than in the AC. The main negative deflection occurred at 39, 37, and 35 ms in the HP, AMY, and PFC, respectively, and the main positive deflection was at 65, 65, and 75 ms. We then calculated the T/C ratio of the main negative deflection (N35) and positive deflection (P70). The T/C ratios of N35 were 0.61, 0.50, and 0.25 in the HP, AMY, and PFC, respectively, and the T/C ratios of P70 were 0.51, 0.35, and 0.28, respectively. The decrease in the T/C ratio reflected an increase in IG effect. However, none of the T/C ratios were obviously changed by anesthesia (0.58, 0.52, and 0.21 for N35; 0.45, 0.30, and 0.23 for P70).

**Figure 9:**
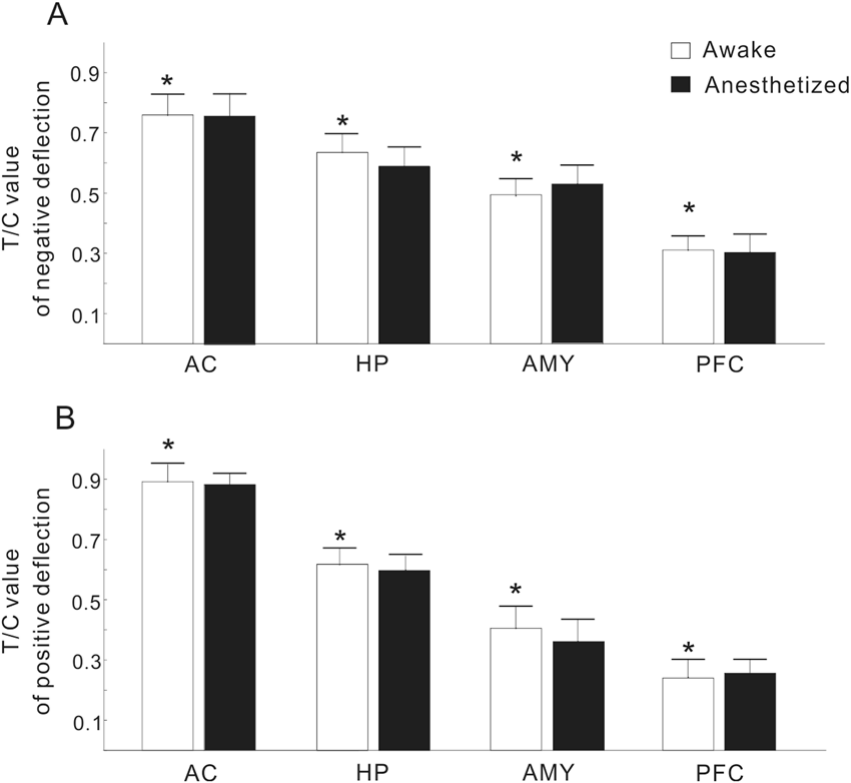
T/C values of negative (**A**) and positive deflection (**B**) under conscious and anesthetized conditions in different brain regions. The data indicate that the degrees of IG are different among different brain regions, but similar between conscious and anesthetized conditions. The data are expressed as the mean ± SE; *Represents a significant difference between any pairs of comparison of T/C values under conscious conditions (P < 0.05, ANOVA with Tukey’s post hoc test).

The mean and SE of the T/C ratios in each brain region are illustrated in Fig. 9. Consistent with the observation from the individual examples, the T/C ratio gradually decreased from the AC, to the HP, AMY, and PFC. There was a significant difference between different brain regions (ANOVA and Tukey’s multiple comparison, p < 0.05). However, no significant difference between the conscious and anesthetized conditions (paired t-test, p > 0.05) could be found in any brain regions.

## Discussion

The primary goal of this study was to investigate the effects of general anesthesia on the widely used electrophysiological examinations: ASSR and IG. We used microwires implanted into different brain areas to record sound-evoked LFPs, which can provide more accurate information from distinct functional areas. Our results showed that the degree of ASSR and IG varied among the AC, HP, AMY, and PFC. More importantly, we found that anesthesia had different effects on ASSR and IG.

### ASSR in different brain areas

ASSR can serve as a useful tool in testing the functional state of the networks supporting neural synchrony^1,2^. The MTP and PLF of 40-Hz ASSR are well used in clinical examinations^5,8,25^. However, the generation source of ASSRs remains unclear, because most ASSRs in clinic are recorded from the human scalp using electrodes for EEG recording. The results of previous PET and fMRI studies suggest that ASSR may be generated by a widely distributed network of sources, located in cortical as well as subcortical regions^25,26^. In this study, we used electrodes which directly penetrated into the brain, and recorded the LFPs in both auditory and non-auditory centers. We selected the HP, AMY, and PFC because they play vital roles in advanced brain functions (memory, emotion, and motivation) and are involved in mental disorders. We found that the AC, as the primary center receiving auditory input, showed the strongest ASSR, the HP and AMY also showed a robust ASSR, but the PFC had no ASSR. This result indicates that the neural network from the auditory system also participates in ASSR. Further studies are needed to clarify whether the HP and AMY directly contribute to the generation of ASSR or just receive the ASSR spread from the auditory pathway.

### Anesthetic effect on ASSR

Previous studies on surgical patients reported that the amplitude of the 40-Hz ASSR was attenuated or possibly abolished with a loss of consciousness after the application of anesthetics (thiopental, fentanyl, and isoflurane)^27–30^. Thus, it was proposed that the amplitude of the ASSR may be a reliable indicator of the level of consciousness^28^. Furthermore, studies on the effects of subanesthetic ketamine demonstrated that the amplitude of the 40-Hz ASSR was augmented by administering a low dose of ketamine, which could not cause a loss of consciousness in most human and animal subjects^31,32^. In this study, we used chloral hydrate to produce a general anesthesia in the rats. We only recorded the LFPs when the rats were completely unconscious, because hyperactivity during light anesthesia and the recovery period made it difficult to stably record the LFPs. Consistent with previous reports, we found that both the MTP and PLF of 40-Hz ASSR were significantly reduced by chloral hydrate induced anesthesia.

Regarding underlying mechanisms, many reports have documented that chloral hydrate potentiates the function of GABAA receptors^33,34^. Parvalbumin inhibitory interneurons expressing GABAA receptors have been proven to play an important role in neural synchronization in the gamma frequency range (30–80 Hz)^35^. Thus, the reduction in the 40-Hz ASSR may be due to the potentiation of inhibitory GABAA function induced by chloral hydrate. Alternately, chloral hydrate may also suppress the transmission of NMDA receptor-mediated glutamate^34,36^, which is expressed on principal excitatory neurons and critical for ASSR. The detailed cellular mechanisms are worthy of further investigation.

### IG in different brain regions

In this study, we found an obvious IG effect in both the AC and in non-auditory regions. When two auditory stimuli were delivered 500 ms apart, at least some reduction in the amplitude of the response to the second stimulus was observed at all sites. However, there were clear differences in the amount of this suppression, or gating, between brain regions. The HP, AMY, and PFC, all components of the non-auditory circuit, showed significantly greater gating than did the AC. Previous electrophysiological studies on rats reported robust auditory-evoked potentials and IG in the HP^37^, AMY^38^, and PFC^11^. The exact anatomical sites involved in mediating sensory gating in humans have not yet been established due to the limitations in performing studies using invasive intra-cerebral electrodes. However, several studies using intracranial electrodes in patients undergoing invasive pre-surgical evaluation have showed that the HP and PFC contribute to the generation of IG^39–41^. Functional imaging studies on humans have also suggested involvement of the HP and PFC in the IG process^42^. All these results support the hypothesis that IG of auditory-evoked responses originates in the non-auditory circuit and not in the AC. In contrast, ASSR may mainly originate from the auditory pathway. Thus, our findings suggest that IG is more suited to examining the function of non-auditory regions related to high order functions (memory, emotion, and cognition).

### Anesthetic effect on IG

The most surprising result of this study is that IG in all the tested regions was little affected by chloral hydrate anesthesia. The exact neural mechanism responsible for IG is not fully understood, but was proposed to be regulated by recurrent inhibitory and excitatory circuits involving multiple neurotransmitter systems^43–46^. GABAergic neurotransmitter systems, as primary inhibitory mechanisms, are thought to modulate the reduction in the excitatory response to the T stimulus^47,48^. Though chloral hydrate has an enhancive effect on the GABAergic inhibitory mechanism^33,34^, it can also suppress glutamatergic transmission^34,36^, which is postulated to produce the excitatory responses to the C stimulus^45,46^. Thus, as both T-amp and C-amp were reduced by chloral hydrate, the T/C ratio was less changed. It should be noted that other neurotransmitters, such as serotonin^49,50^, noradrenaline^51,52^, and acetylcholine^53,54^ are also expected to play a part in the IG process. The interactions between chloral hydrate and these neurotransmitter systems remains unknown. Furthermore, whether other anesthetics have similar effects on IG warrants future investigation.

In conclusion, we found that ASSR was more prominent in the auditory region, while IG was more obvious in the non-auditory regions. ASSR was obviously reduced by general anesthesia, while IG was not. These findings provide a valuable reference for the choice of electrophysiological examinations used in clinical settings.
